# Redifferentiation Therapies in Thyroid Oncology: Molecular and Clinical Aspects

**DOI:** 10.3390/jcm13237021

**Published:** 2024-11-21

**Authors:** Petra Petranović Ovčariček, Murat Tuncel, Atena Aghaee, Alfredo Campennì, Luca Giovanella

**Affiliations:** 1 Department of Oncology and Nuclear Medicine, University Hospital Center Sestre Milosrdnice, 10 000 Zagreb, Croatia; 2 School of Medicine, University of Zagreb, 10 000 Zagreb, Croatia; 3 Department of Nuclear Medicine, Hacettepe University, 06230 Ankara, Turkey; murat.tuncel@hacettepe.edu.tr; 4 Nuclear Medicine Research Center, Mashhad University of Medical Sciences, Mashhad 9177948564, Iran; aghaeeat@gmail.com; 5 Unit of Nuclear Medicine, Department of Biomedical and Dental Sciences and Morpho-Functional Imaging, University of Messina, 98100 Messina, Italy; acampenni@unime.it; 6 Department of Nuclear Medicine, Gruppo Ospedaliero Moncucco, 6900 Lugano, Switzerland; luca.giovanella.md@gmail.com; 7 Clinic for Nuclear Medicine, University Hospital and University of Zurich, 8004 Zurich, Switzerland

**Keywords:** differentiated thyroid cancer, radioactive iodine refractory, redifferentiation, tyrosine kinase inhibitors, BRAF, MEK, NTRK, RET

## Abstract

Since the 1940s, 131-I radioiodine therapy (RIT) has been the primary treatment for metastatic differentiated thyroid cancer (DTC). Approximately half of these patients respond favorably to RIT, achieving partial or complete remission or maintaining long-term stable disease, while the other half develop radioiodine-refractory DTC (RAI-R DTC). The main genomic alteration involved in radioiodine resistance is the activated mitogen-activated protein kinase (MAPK) pathway, which results in the loss of sodium iodide symporters (NIS). Therefore, RAI-R DTC requires alternative treatment options such as tyrosine kinase inhibitors. Over the past decade, several studies have investigated pharmacological induction or enhancement of NIS expression through “redifferentiation” therapies, mainly targeting the MAPK pathway. These novel approaches can restore radioiodine sensitivity in previously refractory patients and, therefore, potentially reestablish the efficacy of RIT. This review discusses various redifferentiation strategies, including their molecular mechanisms and clinical implications.

## 1. Introduction

The management of RAI-R DTC represents a clinical challenge, demanding a balance between timely intervention and avoiding premature cessation of potentially beneficial treatments. RAI-R DTC presents as clinically heterogeneous, ranging from stable to rapidly progressing disease. This variability underlines the importance of accurately identifying radioiodine refractoriness, a key to initiating appropriate therapy at the optimal time. Given the limited treatment options, it is crucial to avoid prematurely discontinuing 131-I therapy without proper justification.

**Figure 1:**
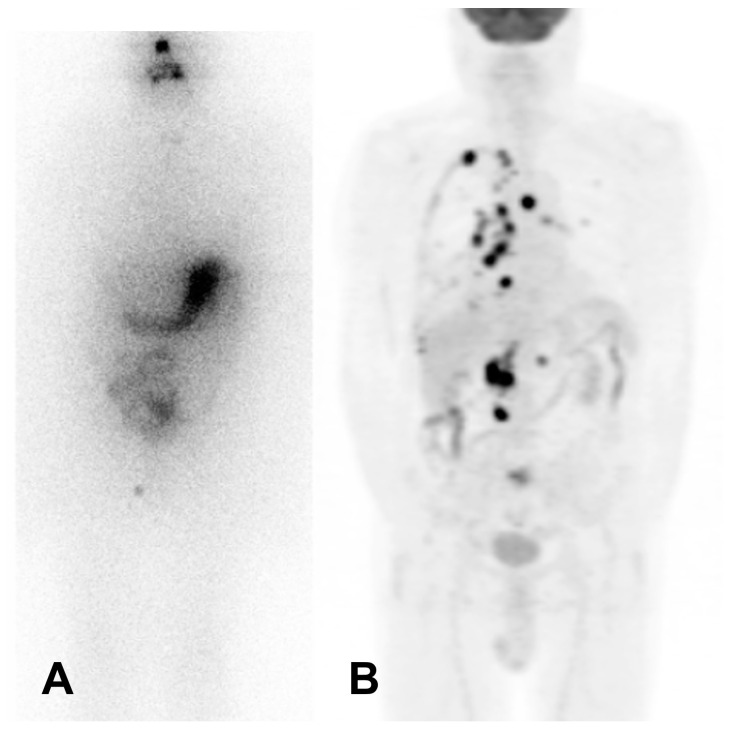
Patient with negative post-treatment whole-body radioiodine scintigraphy (**A**) and multiple [^18^F]FDG-avid lesions (**B**). Legend: male, 67 years, affected by papillary thyroid carcinoma with increasing thyroglobulin level 2 years after thyroidectomy and radioiodine therapy [4.4 GBq]. (**A**) Post-treatment whole-body scintigraphy [7.4 GBq] showing no radioiodine-avid lesions. (**B**) [^18^F]FDG PET/CT: multiple [^18^F]FDG-avid lesions in the mediastinum and lung, with an associated [^18^F]FDG-active pleural effusion (right side), and [^18^F]FDG-avid lesions in the retroperitoneal region. A biopsy on an abdominal lesion confirmed metastasis of papillary thyroid carcinoma.

The definition of RAI-R DTC has been debated within the medical community. The inhibition of NIS expression impairs the ability of DTC cells to accumulate radioiodine, making them refractory to this standard radioiodine treatment. The loss of radioiodine avidity is often associated with other features of dedifferentiation, such as changes in cell morphology and altered expression of thyroid-specific genes. Various criteria have been proposed to identify RAI-R disease. Ten years ago, Schlumberger et al. suggested discontinuing RIT when at least one DTC lesion becomes radioiodine-negative and progresses in size [1]. In the same year, Sacks and Braunstein proposed ceasing RIT in case of negative diagnostic radioiodine scintigraphy in the presence of known structural disease, [^18^F]FDG-positive lesions, or cumulative 131-I activities > 22 GBq [2]. The first and third criteria are highly controversial and largely debated in the literature. Radioiodine uptake on diagnostic radioiodine scan is often considered inadequate since some lesions may show uptake only with high radioiodine activities applied, i.e., on a post-therapy radioiodine scan [3]. Furthermore, in addition to radioiodine uptake in DTC lesions, its response to previous radioiodine treatments and time to progression should be considered when another radioiodine therapy is indicated rather than just cumulative activity applied, especially during a long-term period. Progression is commonly considered if it develops during 6–12 months following RIT [3]. [^18^F]FDG uptake is considered useful in classifying disease as RAI-R. According to a prospective study by Li et al., the FDG SUVmax cut-off of four predicts RAI-R disease with sensitivity, specificity, positive predictive value, and negative predictive value of 75.3%, 56.7%, 76.1%, and 54.8%, respectively [4] (Figure 1).

In 2015, the American Thyroid Association defined RAI-R disease as DTC lesions outside the thyroid bed not accumulating radioiodine on the first post-therapeutic whole-body scan (WBS), lesions that previously accumulated radioiodine but subsequently lost uptake, mixed radioiodine uptake (some lesions radioiodine-positive, others radioiodine-negative), and disease progression despite significant radioiodine uptake [5]. Aggressive variants of papillary thyroid carcinoma (e.g., tall cell, diffuse sclerosing, and hobnail) and mutations such as BRAF V600E and TERT promoter are also associated with increased tumor aggressiveness and RAI-R disease [6,7].

It is important to note that standardization in preparation for radioiodine scans, administered activity, and imaging protocols, including single-photon emission computed tomography/computed tomography (SPECT/CT), is crucial to avoid false-negative scans and ensure the continuation of potentially beneficial RIT [8].

In a joint statement, the American Thyroid Association, European Association of Nuclear Medicine, Society of Nuclear Medicine and Molecular Imaging, and European Thyroid Association acknowledged that the current criteria for defining RAI-R disease are inadequate and should not be considered absolute determinants for recommending or withholding RIT [9].

**Figure 2:**
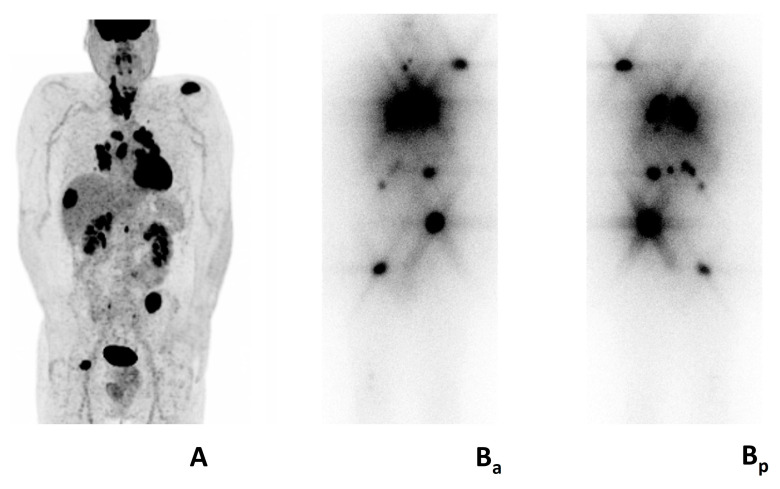
Mixed metabolic features in patient with metastatic follicular thyroid carcinoma. Multiple [^18^F]FDG avid (**A**) and radioiodine avid lesions (**Ba**, anterior; **Bp** posterior). Legend: male, 48 years, referred to the emergency room for intense pain corresponding to the right scapula. Multiple bone, lymph node, lung, liver, and renal metastasis at computed tomography. Bone biopsy (right scapula): follicular thyroid carcinoma. (**A**) preoperative [^18^F]FDG PET/CT: gross involvement of the thyroid gland and multiple intensely [^18^F]FDG-avid metastasis. Post-treatment whole body scan (**Ba**, anterior; **Bp**, posterior): intense radioiodine-avidity of most [^18^F]FDG-avid metastases.

RIT is stopped once RAI-R disease is confirmed and alternative therapeutic options are considered. However, it is crucial to note that RAI-R disease can remain asymptomatic and indolent for years, not always requiring immediate therapy. These patients typically undergo thyroid-stimulating hormone (TSH) suppression and active surveillance that includes measurement of serum thyroglobulin (Tg) and Tg antibodies, conventional cross-sectional imaging (i.e., computed tomography, CT, magnetic resonance imaging, MRI, and [^18^F]FDG positron emission tomography/computed tomography, PET/CT) every 3–6 months [5,6,10]. During surveillance, disease progression may show heterogeneous patterns, with some lesions progressing while others remain stable or have mixed metabolic features at molecular imaging (Figure 2).

At this point, localized treatments such as surgery, external beam radiation therapy, thermal ablation, or stereotactic radiosurgery may be employed. In cases of significant, multi-lesional progression that cannot be managed with localized therapies, systemic treatment with tyrosine kinase inhibitors is usually initiated [10].

**Figure 3:**
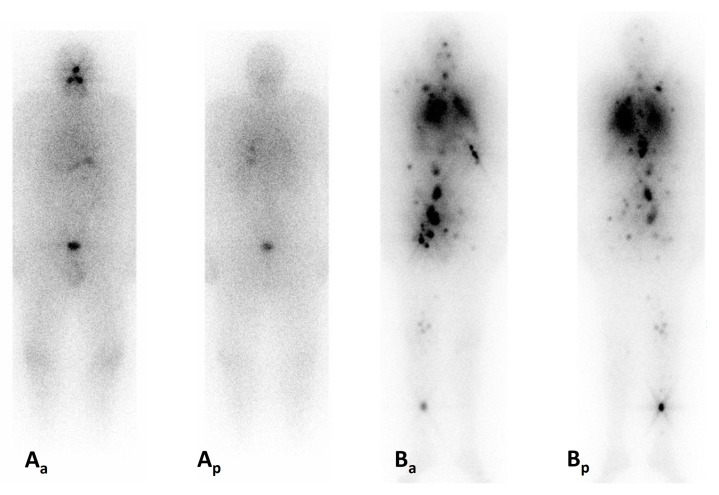
Redifferentiation of radioiodine-refractory disease. **Legend:** Male, 63 years, affected by a papillary thyroid carcinoma [pT3a(m), pN1b, pMx] developed multiple lung and bone metastasis without iodine-uptake whole-body scintigraphy 5 days after administration of 7.4 GBq I-131 (**Aa**, anterior; **Ap**, posterior). An off-label treatment with dabrafenib (150 mg/2xday) and trametinib (2 mg/day) was started after approval from the insurance company. Two months later, 7.4 GBq of I-131 were administered, and the post-treatment whole-body scintigraphy (**Ba**, anterior, **Bp**, posterior) showed multiple, intensely radioiodine-avid lesions at the level of the lymph nodes, lungs, and musculoskeletal metastases. Six months after treatment, a partial biochemical response was recorded with a thyroglobulin decrease from 2276 to 825 μg/L.

Several studies have focused on strategies to reinduce or enhance radioiodine uptake in RAI-R disease, potentially reestablishing radioiodine sensitivity and enabling the de novo application of radioiodine in previously RAI-R DTC patients (Figure 3).

## 2. Redifferentiation Concepts

The concept of reinducing or enhancing NIS expression for RIT is in line with personalized therapeutic principles that offer a tailored approach to treating RAI-R DTC. Early attempts to redifferentiate metastatic RAI-R DTC using retinoic acid and PPAR-γ agonists, for example, demonstrated low efficacy. However, more recent advances in understanding the molecular pathways of RAI-R disease have enabled more focused research with promising results [7].

In vitro studies have shown a crucial relationship between the MAPK pathway and NIS expression [11,12]. The MAPK pathway, which is also known as the RAS-RAF-MEK-ERK pathway, is a signaling molecular cascade involved in cell proliferation and differentiation. Stimulation of the MAPK pathway via MEK and ERK activation is associated with reduced NIS expression at transcriptional and post-transcriptional levels. The activated MAPK pathway is commonly noted in DTC harboring mutations in BRAF, RAS, or RET genes, which links genetic alterations and radioiodine refractoriness. BRAF mutations are present in approximately 50% of papillary thyroid cancers (PTC) and are strongly associated with the inhibition of NIS expression and the development of RAI-R disease [13]. RET mutations, which are commonly detected in PTC, and RAS mutations, frequently noted in follicular thyroid cancer (FTC), are also involved in MAPK pathway activation [7]. Moreover, a positive correlation has been demonstrated between the degree of DTC dedifferentiation and its loss of NIS expression with the level of MAPK pathway activation. This relationship seems to be hierarchical, with BRAF V600E mutations associated with more enhanced effects than RAS or RET mutations [14]. This hierarchy may result from varying degrees of MAPK pathway activation linked with each mutation. The loss of NIS expression and radioiodine avidity often occurs continuously during a certain period rather than instantly, which correlates with the progressive activation of the MAPK pathway. It may explain why some DTCs retain partial radioiodine sensitivity while others become entirely refractory. Even within a single patient, different metastatic lesions may have varying degrees of MAPK pathway activation and, consequently, different levels of radioiodine avidity. Understanding this molecular basis of RAI-R DTC provides a solid basis to targeting the MAPK pathway as a potential strategy for redifferentiation [15].

Preclinical studies using animal models demonstrated that inhibiting activated intracellular signaling pathways could lead to the re-expression of NIS on the DTC cell membrane [16,17]. By reactivating the cellular mechanisms responsible for iodine uptake, redifferentiation therapy has the potential to reestablish the effectiveness of RIT in patients who have become unresponsive.

Recently, Aghaee and colleagues reported a case of a RAI-R patient with lung and bone metastases that restored RAI-avidity after treatment with receptor radioactive therapy (PRRT) with [^177^Lu]Lu- DOTA-TATE [18].

The biological basis of this phenomenon is not known, but the possibility of sequential nuclear medical therapy introduces a fascinating perspective. Naturally, the phenomenon will have to be replicated, and currently, no clinical studies are available.

## 3. Clinical Studies

The first clinical trial conducted at Memorial Sloan Kettering Cancer Center, published in 2013, was a significant step forward in redifferentiation treatments [19]. This study demonstrated that targeted MEK inhibitor therapy could effectively reinduce 131-I uptake in previously RAI-R tumors. The trial enrolled 24 patients with RAI-R DTC, each presenting with at least one radioiodine-negative lesion on 124-I PET/CT scans and radiographically progressive disease within the previous 12 months. Participants received selumetinib, a MEK1/2 inhibitor, at 75 mg twice daily for four weeks. Following treatment, 20 patients underwent follow-up 124-I PET/CT scans after recombinant human TSH (rhTSH) administration. The study demonstrated that 60% of evaluated patients (12 out of 20) showed restored radioiodine uptake. Subsequent lesion-specific dosimetry identified eight patients who met the threshold for RIT, defined as a dose of ≥20 Gy with a 131-I administered activity ≤ 11.1 GBq. These patients received RIT, resulting in five partial responses (PR) and three cases of stable disease (SD) over a six-month follow-up period. Notably, all treated patients showed a reduction in DTC lesion size. The treatment was mainly well-tolerated, with rash, fatigue, and edema being the most common side effects. More severe adverse events were rare, with one case each of grade 3 pain and increased liver enzymes, and no grade 4 or 5 events reported. Interestingly, the study revealed a potential correlation between genetic mutations and treatment response. Patients with NRAS mutations demonstrated a higher response rate compared to those with BRAF mutations, i.e., all five patients with NRAS mutations showed restored radioiodine uptake and received RIT. On the other hand, only four out of nine patients with BRAF mutations restored radioiodine uptake, with just one reaching the dosimetry threshold for RIT. The remaining two treated patients had RET-rearrangements and wild-type PTC, respectively. This study provided the first prospective evidence that targeted therapy could restore radioiodine avidity in RAI-R DTC. It also underlined the importance of tumor genotype in predicting response to redifferentiation therapy, particularly the high response rate observed in NRAS-mutant tumors. The differential response between NRAS and BRAF mutant tumors was suggested to be due to insufficient blockade of the MAPK pathway with MEK inhibitors in BRAF-mutant tumors. This finding encouraged further investigation with specific BRAF inhibitors such as dabrafenib and vemurafenib. These therapies were subsequently evaluated as monotherapies or in combination with MEK inhibitors in additional clinical trials focused on BRAF-mutated RAI-R disease.

Rothenberg and colleagues published a study on the efficacy of BRAF inhibition in patients with BRAF V600E-mutant RAI-R DTC two years later [20]. This targeted approach addressed the previously observed findings related to BRAF-mutant tumors. The study enrolled ten patients, each receiving a four-week course of the BRAF inhibitor dabrafenib at a dose of 150 mg twice daily. Following this initial treatment period, patients underwent a 131-I WBS after rhTSH stimulation to assess changes in radioiodine uptake. Six out of ten patients exhibited new sites of radioiodine uptake on diagnostic 131-I WBS. For these responders, the dabrafenib therapy was extended for an additional two weeks, followed by a fixed empiric activity of 5.5 GBq of 131-I. Two patients demonstrated PR at the three-month follow-up, while four maintained SD. Importantly, the treatment regimen was generally well-tolerated. Most detected adverse events were mild to moderate (grade 1 or 2). However, one patient developed a grade 3 squamous cell carcinoma of the skin, a well-known side effect associated with BRAF inhibitors. This lesion was successfully managed through excision with clear margins, underlining the importance of careful patient monitoring during redifferentiation therapies.

The results of this study were encouraging, regarding the restored radioiodine uptake and clinical outcomes in BRAF-mutated RAI-R DTC. The findings suggested a potential advantage of BRAF inhibitors over MEK inhibitors in BRAF-mutated tumors.

Jaber et al. conducted a retrospective study of 13 patients with RAI-R thyroid cancer who were treated with either single-agent or combination MEK/BRAF inhibitors and underwent 131-I diagnostic WBS during treatment [21]. Among the study population, nine patients harbored BRAF V600E mutations, three had NRAS mutations, and one patient had wild-type thyroid cancer. The median duration of targeted therapy before diagnostic WBS was 14.4 months, ranging from 0.9 to 76.4 months. Initial evaluation revealed that eight patients demonstrated restored 131-I uptake and subsequently received a median activity of 7.4 GBq of 131-I. One additional patient was treated empirically despite no clear evidence of restored 131-I uptake. Of note, only three patients underwent dosimetric pre-therapeutic evaluation, while the remaining patients received empirically determined 131-I activities. Prior to 131-I treatment, eight patients had SD disease, and one patient showed progressive disease (PD). Following 131-I treatment, with a median follow-up of 14.3 months, three patients achieved PR while six maintained SD, including the empirically treated patient.

In another study at Memorial Sloan Kettering Cancer Center, Dunn and colleagues further evaluated the potential of BRAF inhibition in RAI-R DTC [22]. This research focused on vemurafenib, another BRAF inhibitor, as a redifferentiation therapy for patients with BRAF-mutated RAI-R DTC. The study enrolled 12 patients, administering vemurafenib (960 mg twice daily) for four weeks. Of the initial cohort, ten patients completed the whole treatment cycle. Following the vemurafenib regimen, patients underwent a 124-I PET/CT scan with rhTSH stimulation to assess changes in radioiodine uptake and perform lesional dosimetry. The criterion for subsequent RIT was at least one radioiodine-negative lesion ≥ 5 mm in diameter, achieving a target dose of ≥20 Gy with calculated administered 131-I activity of ≤11.1 GBq. After redifferentiation therapy, the 124-I PET/CT demonstrated new or increased radioiodine uptake in six out of ten patients. Four out of six patients met the dosimetry threshold criteria and underwent RIT. At the six-month follow-up, two patients achieved PR, while two maintained SD. In general, the treatment was well-tolerated, with common side effects including rash, fatigue, nausea, and arthralgia. Importantly, no grade 4 or 5 adverse effects were observed.

Interestingly, molecular studies conducted on tumor biopsies from three patients provided valuable findings into the mechanism of action. The data revealed an increased thyroid differentiation score, a lower mitotic index, and higher NIS mRNA levels. These molecular changes corroborated the clinical findings and suggested that vemurafenib promoted redifferentiation of the RAI-R cancer cells.

Weber et al. conducted another prospective study (ERRITI) investigating the efficacy of genotype-guided MAPK inhibition redifferentiation therapy in RAI-R DTC [23]. The study included twenty patients: six with BRAF-mutated tumors that received a combination of dabrafenib in a dose of 75 mg twice daily and trametinib 2 mg daily, while fourteen with BRAF wild-type tumors received trametinib alone (2 mg daily) for 21 days. Post-treatment, patients underwent 123-I SPECT/CT imaging. Redifferentiation was considered successful if a target-to-background ratio > 4, and a 2-fold higher uptake than mean liver uptake was detected in at least one tumor lesion. This criterion was met in seven patients (two BRAF-mutated, five BRAF wild-type), indicating similar redifferentiation rates regardless of BRAF mutation status. All responding patients received personalized, dosimetry-guided RIT, taking into account activity in the lungs, blood, and individual tumor lesions. Patients received a mean (range) activity of 300.0 (273.0–421.6) mCi of 131-I therapy. Any thyroglobulin decline was seen in 57% (4/7) of the patients. Peak standardized uptake value (SUVpeak) < 10 on [^18^F] FDG PET was linked with successful redifferentiation (*p* = 0.01). One-year follow-up revealed RECIST 1.1 PR in one patient, SD in five, and PD in one case. The treatment was mainly well-tolerated, with most adverse events (38/40) graded 1 or 2. Common side effects included rash and diarrhea. More severe reactions were rare: one BRAF-mutated patient experienced grade 3 pyrexia, which resolved after a 2-day treatment interruption, and one BRAF wild-type patient developed a transient grade 4 skin rash three days post-therapy completion.

Leboulleux conducted a MERAIODE multicentric prospective phase II trial in patients with RAI-R DTC, with two independent cohorts, one for BRAF V600E and one for RAS mutated DTC. From one of these cohorts, Leboulleux et al. published a prospective phase II multicenter trial focusing on patients with BRAF-mutated RAI-R DTC [24]. The study included twenty-four patients who had experienced RECIST progression within 18 months and had lesions < 3 cm. The treatment protocol consisted of dabrafenib in a dose of 150 mg twice daily and trametinib 2 mg once daily for five weeks, followed by an empiric activity of 5.5 GBq RIT after rhTSH stimulation, regardless of diagnostic 131-I WBS results. Twenty-one patients were evaluated for treatment efficacy at six months. Post-therapeutic WBS showed uptake in twenty patients. The 6-month follow-up revealed PR in eight patients (38%), SD in eleven (52%), and PD in two (10%), with no complete responses (CR). Patients achieving PR at 6 or 12 months were eligible for a second course of targeted therapy. Of the eleven patients undergoing it, there was one CR, six PR, two SD, and one PD at the 6-month follow-up, with one patient not evaluable. The progression-free survival (PFS) rate was 82% at one year and 68% at two years. One patient died due to PD at two years. Most detected adverse events were mild to moderate, with 25% grade 1 and 42% grade 2. Six patients experienced nine grade 3 adverse events (infectious syndrome, psoas hematoma), and there was one grade 4 event (anicteric cholestasis). Common side effects included asthenia, nausea, lymphopenia, fever, diarrhea, fatigue, and skin-related issues. The higher response rates in this study compared to Weber’s may be due to the longer duration of redifferentiation therapy, higher dabrafenib dosage, small tumor foci, and more limited tumor volume. In many patients, a decreased tumor size was already significant at one month of dabrafenib-trametinib treatment, and this clearly raises the question of whether benefits from the treatment are related to the dabrafenib-trametinib treatment or to the addition of 131-I to dabrafenib-trametinib. However, due to the long PFS, and because the 6-month response was independent of the 1-month tumor response, the treatment efficacy was believed to be not only due to dabrafenib-trametinib treatment during the 6 weeks but also to its association with 131-I. As this is a similar issue with other redifferentiation agents, a comparative randomized trial comparing kinase inhibition ± RIT would be the only way to answer this specific question.

In another patient cohort, Leboulleux et al. conducted another study focusing on metastatic RAI-R RAS-mutated DTC [25]. The study investigated the efficacy of trametinib treatment followed by 131-I administration after rhTSH stimulation. Eleven patients were initially enrolled, with ten evaluated six months after the first treatment course. The protocol consisted of daily 2 mg trametinib for seven weeks, with a fixed 131-I activity of 5.5 GBq administered on day 35 (±2 days), regardless of diagnostic 131-I WBS results. Six months after the initial treatment, there were two PR (20%), seven cases of SD (70%), and one PD. Patients achieving PR at 6 or 12 months were eligible for a second course of trametinib and 131-I therapy. Of the three patients who received this second course, one maintained PR for 18 months, while two experienced PD at 3- and 6-months post-treatment. The median progression-free survival was one year. Adverse events occurred in nine out of eleven patients (82%), predominantly grade 1 (36%) and grade 2 (27%). Two patients experienced grade 3 adverse events, with no grade 4 events reported. Treatment discontinuation was necessary in two cases due to grade 3 erythematous colitis and grade 2 decrease in left ventricular ejection fraction. The six-week trametinib therapy increased 131-I uptake in two-thirds of RAI-R RAS-mutated DTC patients. However, the treatment’s efficacy was limited, with only a 20% response rate at six months. These results suggest that while the approach shows some promise in restoring radioiodine uptake, its overall effectiveness in managing this specific subtype of thyroid cancer is suboptimal.

Iravani et al. treated patients with tumors harboring an NRAS mutation with a MEK inhibitor (trametinib) and tumors with a BRAF V600E mutation with combined BRAF and MEK inhibition (dabrafenib and trametinib; or vemurafenib and cobimetinib) for four weeks. Six patients received redifferentiation therapy. Three patients had an NRAS mutation, two with FTC and one with a poorly differentiated thyroid carcinoma, and three patients had a BRAF V600E mutation and PTC. One NRAS and all BRAF V600E mutation cases demonstrated restoration of RAI uptake and proceeded to RAI therapy with a median follow-up of 16.6 months (range 13.5–42.3 months). The patient with an NRAS mutation and two of three patients with a BRAF V600E demonstrated partial imaging response beyond a three-month follow-up [26].

Burman et al. conducted a phase 2 trial evaluating trametinib’s efficacy in RAI-R DTC patients with RAS mutations and wild-type RAS [27]. If the post-therapy 124-I PET/CT demonstrated increased radioiodine uptake, allowing a target dose of ≥20 Gy with a calculated 131-I activity of ≤11.1 GBq, patients received RIT according to whole body and blood dosimetry. In the RAS-mutant group of twenty-five patients, fifteen met the dosimetry threshold for RIT based on 124-I PET/CT imaging. Fourteen of these patients received RIT. The 6-month follow-up revealed PR in eight patients (57%), SD in three patients (21%), and PD in three patients (21%). The PFS rate at 6 months was 44% for the RAS-mutant group. The RAS wild-type group consisted of nine patients, including those with BRAF mutations, RET alterations, and one case of STK11 mutation. In this cohort, three out of four patients with BRAF mutations and one out of four patients with RET alterations met the dosimetry threshold for RIT. Treatment outcomes for the RAS wild-type group showed three cases of SD and one PR, with the PR observed in a patient with a BRAF-mutated tumor.

This study again demonstrates varying degrees of treatment efficacy across different genetic profiles in RAI-R DTC, with RAS-mutant patients showing a significant response to the trametinib-RIT combination therapy. Redifferentiation therapy has shown promising results beyond tumors with BRAF and RAS mutations, demonstrating potential efficacy in thyroid cancers with various other genetic alterations.

Groussin et al. reported a successful case of redifferentiation therapy using larotrectinib in a patient with RAI-R DTC characterized by an EML4–NTRK3 gene fusion [28]. However, similar to BRAF mutations, the co-occurrence of TERT mutations and NTRK fusions may also contribute to re-sensitization failure [29].

Similarly, encouraging results have been observed in patients with RET rearrangement RAI-R DTC. Specifically, two RET inhibitors, pralsetinib [30] and selpercatinib [31], have both been reported to re-sensitize RET rearrangement-positive tumors to RIT. Recently, Werner et al. used a successful combination of selpercatinib and radioiodine after pretherapeutic dose estimation in RET-altered RAI-R DTC. Upon disease progression, the patient received the selpercatinib. A diagnostic 131-I WBS was conducted after 15.5 months of RET inhibitor therapy, showing intense radiotracer accumulation in sites of disease. After RIT (9.4 GBq), previously negative lung nodules showed intense radiotracer accumulation on post-therapeutic scans, followed by a decrease in Tg levels and nodule size on CT. This individualized approach allowed the administration of substantially higher activities (achieving tumor doses of 197 Gy) [32].

Similar to BRAF inhibitors, as the RET inhibitors induce redifferentiation, I-131 uptake increases, and [^18^F]FDG uptake decreases [30].

In two distinct cases, comprehensive genomic profiling revealed the presence of specific fusion oncogenes: TPR-NTRK1 and CCDC6-RET [31]. Considering these gene alterations, a combined approach using two targeted therapies (larotrectinib, an NTRK inhibitor, and selpercatinib, a RET inhibitor) was implemented, which resulted in decreased tumor size and reinduced RAI uptake.

Toro-Tobon et al., in a retrospective study, evaluated thirty-three patients with progressive metastatic RAIR-DTC who underwent redifferentiation therapy at Mayo Clinic between 2017–2022 [33]. Depending on genetic alterations, patients received MEK, RET, or ALK inhibitors alone or BRAF-MEK inhibitor combinations for four weeks, with high-activity 131-I therapy administered to those showing increased RAI avidity at week three. Radioiodine uptake was restored in 57.6% of patients. Among PTCs, 42.1% showed restoration, while all invasive encapsulated follicular variant PTCs and FTCs demonstrated restored uptake. Notably, all RAS mutant tumors responded to redifferentiation, compared to 38.9% of BRAF mutant cases. Both redifferentiated and non-redifferentiated groups showed similar clinical outcomes, with approximately 12% tumor shrinkage at three weeks. The redifferentiated group achieved an additional 20% tumor reduction at six months follow-up. No significant differences were observed in PFS or time to additional therapy between groups. Among observed adverse outcomes, anaplastic transformation occurred in 6.1% (2/33) of patients. Five patients (15.1%) died during follow-up, with all deaths occurring in patients who had undergone both redifferentiation and subsequent 131-I therapy. Redifferentiation therapy showed particular promise in RAS-driven follicular phenotypes, though further research is needed to evaluate survival outcomes and potential risks of anaplastic transformation following high-activity RIT.

Table 1 summarizes different redifferentiation studies and their protocols.

**Legend**: PET, positron emission tomography; BRAF-WT, BRAF-wild type; BRAF-MUT, BRAF-mutated; TSH, thyroid stimulating hormone; p.o., per os; q.d. quaque die; bid, *bis in die.*

**Figure 4:**
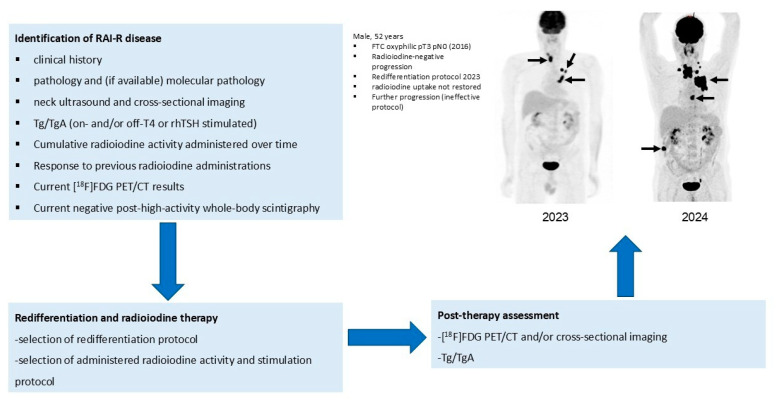
Clinical flowchart in redifferentiation therapies. **Legend**: FTC, follicular thyroid carcinoma; Tg, thyroglobulin; TgA, thyroglobulin antibodies; T4, thyroxine, rhTSH, recombinant human thyroid-stimulating hormone; PET/CT, positron-emission tomography.

An example of the clinical flowchart in redifferentiation therapies is shown in Figure 4.

## 4. Second-Line TKI-Related Adverse Effects

The side effects related to the use of first-line TKI drugs are well known since they are widely reported in the literature, also highlighting their different prevalence according to the use of lenvatinib or sorafenib [34,35].

However, the use of second-line TKI drugs, either BRAF or MEK inhibitors or both (e.g., dabrafenib or trametinib), is also associated with numerous adverse effects having a negative impact on patients’ quality of life, necessitating dosage reductions in nearly two-thirds of patients or treatment withdrawal in up to 20% of patients, while therapy-related death was reported in 1.5–2% [26].

Recently, some authors reported on the most common treatment-related adverse effects due to the use of a BRAF inhibitor alone or in combination with a MEK inhibitor, highlighting their different prevalence [36].

Table 2 summarizes the most common adverse effects due to the use of second-line TKI agents either as single-line therapy (i.e., using BRAF or MEK inhibitors) or in combination (i.e., using BRAF and MEK inhibitors)

Finally, it is important to consider in the clinical management of RAI-R disease that some patients can develop TKI resistance, thus worsening their outcome [26].

Accordingly, the ability of these agents to restore or enhance radioiodine uptake in such patients, offering the possibility of a de novo application of radioiodine therapy, should always be taken into account as soon as possible, also to reduce the risk of adverse effects (especially if severe) that could cause discontinuation (temporarily or permanently) of their use.

## 5. Current Paradigms and Future Perspectives

Current evidence suggests that specific targeted therapies have the potential to modulate the iodine-handling mechanisms of thyroid cancer cells, effectively restoring or enhancing their ability to accumulate 131-I.

These targeted therapies, based on gene profiling, demonstrate efficacy in two main scenarios [38]:

1. *Re-establishing 131-I uptake*: Targeted therapies can re-induce the expression of radioiodine uptake and retention. This “redifferentiation” effect could potentially convert RAI-R tumors back to a radioiodine-treatable state.
2. *Enhancing existing uptake*: These agents may increase the uptake of 131-I in DTC lesions that still demonstrate some degree of iodine accumulation. Optimizing the cellular pathways responsible for iodine processing could significantly increase the amount of radioiodine concentrated within tumor tissues.

The implications of these effects on treatment outcomes are multifactorial:

1. *Increased tumor radiation absorbed dose*: Enhanced 131-I uptake translates to a higher absorbed radiation dose per unit of administered 131-I activity (Gy/GBq or rad/mCi.
2. *Improved treatment efficacy*: The increased radiation dose to tumor tissues may lead to better clinical outcomes, including partial tumor regression or stabilization of disease.
3. *Potential for dose reduction*: The enhanced uptake efficiency might allow the same therapeutic effect with a lower administered activity of 131-I. This strategy may be especially valuable for patients at higher risk of radioiodine-associated side effects.
4. *Minimization of side effects*: By potentially reducing the total activity of 131-I needed for effective treatment, these agents may help reduce the risk of adverse effects associated with high 131-I activity. These side effects can include salivary gland dysfunction, bone marrow suppression, and a small but non-zero risk of secondary malignancies.
5. *Expanded treatment options*: The redifferentiation therapies could reopen new treatment options for patients previously deemed unsuitable for further 131-I therapy.

These preliminary findings of redifferentiation therapy effectiveness are encouraging. However, further studies are essential to:

1. *Validate uptake restoration and enhancement in different clinical and genomic scenarios*: Larger, well-designed studies are needed to confirm the initial observations on the ability of these agents to modulate radioiodine uptake in diverse patient populations and different tumor types based on gene profiling.
2. *Evaluate long-term safety*: As with any new therapeutic approach, it is important to thoroughly investigate these agents’ long-term safety profile, particularly considering their effects on iodine-concentrating organs.
3. *Optimize treatment protocols*: Research should focus on determining the ideal timing, imaging, dosing, and duration of the therapy in relation to 131-I administration to maximize their beneficial effects while minimizing any potential risks.
4. *Identify predictive biomarkers*: Developing reliable methods to predict which patients are most likely to benefit from redifferentiation therapies could help personalize treatment approaches and avoid unnecessary interventions in non-responders.
5. Compare redifferentiation vs. tyrosine kinase inhibitors.
6. Criteria of therapeutic outcomes for consideration of redifferentiation and 131-I retreatment (Tg response, RECIST response, etc.).
7. Cost-effectiveness.

As this field progresses, it holds the potential to significantly reshape the treatment options in RAI-R DTC.

In addition to these specific redifferentiation therapies, several SPECT and PET radiopharmaceuticals offer promising fields for research. Tracers targeting prostate-specific membrane antigens, somatostatin receptors, or fibroblast activation proteins can help determine different molecular features of RAI-R disease. Novel theranostic strategies that extend beyond traditional radioiodine-guided therapies may potentially broaden the therapeutic options for patients with RAI-R thyroid cancer [39]. The application of these diverse radiopharmaceuticals enables a more comprehensive molecular profiling of RAI-R thyroid cancer. This multi-factorial approach can reveal:

1. Heterogeneity within and between primary and metastatic tumors
2. Identify previously unrecognized targets for therapy
3. Guide the selection of the most appropriate treatment strategy for each patient
4. Monitor treatment response at a molecular level.

## 6. Conclusions

Current data suggest that the redifferentiation approach may be adaptable to various genetic profiles in RAI-R DTC, potentially offering new treatment options for patients with diverse molecular subtypes of the disease. Available evidence demonstrates that MAPK pathway blockage is an efficient strategy to redifferentiate some types of RAI-R DTC. This approach is effective both as a single therapy and, more promisingly, as part of a combination treatment regimen. However, several other targets are also available according to gene profiling, with promising results in small studies.

Still, available studies are very heterogeneous in multiple aspects, i.e., definition of RAI-R DTC, the duration of redifferentiation therapy, the imaging modality used to determine restoration of RAI uptake (I-131/I-123/I-124), the applied radioiodine activity, and the method of choice for applied activity—dosimetry-guided or empiric fixed activity. It also remains unclear whether the increase in uptake on diagnostic WBS performed after redifferentiation therapy can serve as a reliable criterion for selecting patients for RAI therapy. To address these uncertainties, additional large-scale multicenter studies are necessary. These studies should aim to identify the optimal choice and duration of redifferentiation therapy, selection criteria for redifferentiation therapy based on genetic testing, characteristics of patients who are the best candidates for this treatment, the risk-benefit ratio of the therapy, and its impact on patient’s quality of life.

## Figures and Tables

**Table 1:** Redifferentiation studies and their protocols.

Study	Type of the Study	Number of Patients	Redifferentiation Agent	Mechanism of Action	Protocol
Ho et al. [19]	Clinical trial	20	Selumetinib	MEK inhibitor	Selumetinib 75 mg p.o. bid for 4 weeks. If the 124-I PET scan indicated increased iodine uptake and delivered a projected absorbed dose of 2000 cGy or more to the lesion, then selumetinib continued to perform dosimetry and was subsequently discontinued 2 days after 131-I therapy with rhTSH stimulation.
Rothenberg et al. [20]	Clinical trial	10	Dabrafenib	BRAF inhibitor	Dabrafenib 150 mg p.o. q.d. for 25 days before the 131-I scan. If positive, dabrafenib was continued for 17 days, therapy with 150 mCi of 131-I was applied, and dabrafenib was continued for five more days.
Jaber et al. [21]	Retrospective	13	Selective trametinib, dabrafenib, and/or vemurafenib	MEK inhibitor, BRAF inhibitor	Patients with BRAF mutation were treated with BRAF inhibitors (7 with dabrafenib, 1 with vemurafenib, and 1 with a combination of dabrafenib/trametinib). Three patients with RAS mutation were treated with MEK inhibitors (two with trametinib and one with an investigational MEK inhibitor). The patient without identified somatic mutations was treated with trametinib
Dunn et al. [22]	Clinical trial	10	Vemurafenib	BRAF inhibitor	124-I PET scan performed before and after Vemurafenib 960 mg p.o. bid for approximately 4 weeks. Those with increased 131-I concentration exceeding a predefined lesional dosimetry threshold were treated with I-131. Vemurafenib was discontinued 2 days after 131-I therapy.
Weber et al. [23]	Prospective study	20	Dabrafenib and Trametinib	BRAF inhibitor and MEK inhibitor	Trametinib 2 mg daily in BRAF-WT for 21 ± 3 days. Trametinib 2 mg trametinib daily + dabrafenib 75 mg twice daily in BRAF-MUT for 21 ± 3 days. Patients in whom the posttreatment 123-I SPECT/CT demonstrated a regional target/background ratio of more than 4 and a 2-fold higher iodine uptake than the mean uptake in liver parenchyma (in at least one tumor lesion) by visual assessment were considered responders, and 131-I therapy was performed
Leboulleux et al. [24]	Clinical trial	21	Dabrafenib trametinib	BRAF inhibitor and MEK inhibitor	Dabrafenib 150 mg p.o. bid and trametinib 2 mg po q.d. for 42 days. On day 28, a radioiodine scan was performed. After 35 days, a therapy of 131-I was administered.
Leboulleux et al. [25]	Clinical trial	10	Trametinib	MEK inhibitor	Trametinib 2 mg po q.d. for 42 days. On day 28, a radioiodine scan was performed. After 35 days, a therapy of 131-I was administered.
Iravani et al. [26]	Retrospective	6	Dabrafenib and Trametinib	BRAF inhibitor and MEK inhibitor	For NRAS: trametinib 2 mg p.o. q.d. 4 weeks For BRAF V600E: combination of dabrafenib 150 mg p.o. q.d. and trametinib 2 mg p.o. q.d. 4 weeks.
Groussin et al. [28]	Case report	1	Larotrectinib	Tropomyosin receptor kinase (TRK) inhibitor	Larotrectinib 100 mg p.o. bid (6 months).
Lee et al. [31]	Two cases	2	Larotrectinib; Selpercatinib	Tropomyosin receptor kinase (TRK) inhibitor; RET inhibitor	Larotrectinib 100 mg p.o. bid; Selpercatinib 80 mg bid.
Werner et al. [32]	Case report	1	Selpercatinib	RET inhibitor	Selpercatinib 160 mg p.o. bid for 3 weeks
Toro-Tobon et al. [33]	Retrospective	33	Trametinib, Selpercatinib, Pralsetinib, Alectinib, Dabrafenib + Trametinib	MEK, RET, or ALK inhibitors alone, or combination BRAF-MEK inhibitors	Redifferentiation therapy for 4 weeks. At the end of week 3, all patients underwent rhTSH-stimulated 123-I WBS. At week 4, those who redifferentiated (any uptake of at least one lesion based on a qualitative assessment) received high-activity 131I therapy.

**Table 2:** Tyrosine kinase inhibitors’ adverse effects.

*Adverse Event*	*Dabrafenib Alone (n = 26)* *Any Grade (%)* *	*Dabrafenib + Trametinib (n = 27)* *Any Grade (%)* *	*Selumetinib + RAI (n = 154)* *Any Grade (%)* **	*Dabrafenib + Trametinib* *(n = 24)* *Any Grade (%)* ***
*Skin/subcutaneous disorders*	17 (65)	9 (33)	69 (45)	-
*Fever*	13 (50)	16 (59)	-	5 (21)
*Hyperglycemia*	12 (46)	5 (19)	-	-
*Anemia*	11 (42)	8 (30)	-	-
*Palmar-plantar erythrodysesthesia syndrome*	11 (42)	6 (22)	-	-
*Nausea*	11 (42)	14 (52)	44 (29)	10 (42)
*Alopecia*	11 (42)	0 (0)	-	-
*Chills*	11 (42)	14 (52)	-	-
*Fatigue*	10 (38)	14 (52)	44 (29)	2 (8)
*Hypophosphatemia*	9 (35)	11 (41)	-	-
*Vomiting*	7 (27)	6 (22)	-	2 (8)
*Rash maculo-papular*	7 (27)	4 (15)	19 (12)	5 (21)
*Weight loss*	7 (27)	0 (0)	-	-
*Anorexia*	6 (23)	9 (33)	-	3 (13)
*Pruritus*	6 (23)	3 (11)	21 (14)	-
*Arthralgia*	6 (23)	0 (0)	-	1 (4)
*Myalgia*	5 (19)	6 (22)	-	-
*Lymphocyte count decreased*	5 (19)	0 (0)	-	3 (13)
*Headache*	5 (19)	0 (0)	8 (5)	2 (8)
*Diarrhea*	4 (15)	7 (26)	68 (44)	5 (21)
*Edema limbs*	3 (12)	5 (19)	30 (19)	-
*Aspartate aminotransferase increased*	0 (0)	10 (37)	-	1 (4)
*Alanine aminotransferase increased*	0 (0)	8 (30)	-	1 (4)
*Alkaline phosphatase increased*	0 (0)	5 (19)	-	-
*Generalized muscle weakness*	0 (0)	5 (19)	-	10 (42)
*Blood creatine phosphokinase increased*	-	-	31 (20)	-
*Hypertension*	-	-	20 (13)	3 (13)
*Stomatitis*	-	-	17 (11)	2 (8)
*Vision blurred*	-	-	16 (10)	-
*Constipation*	-	-	-	2 (8)
*Cough*	-	-	-	1 (4)
*Abdominal pain*	-	-	-	1 (4)
*Bronchitis*	-	-	-	0 (0)
*Hyposialia*	-	-	-	2 (8)
*Leukopenia*	-	-	-	1 (4)
*Neutropenia*	-	-	-	1 (4)
*Thrombopenia*	-	-	-	0
*Vertigo*	-	-	-	1 (4)
*Urinary infection*	-	-	-	0

**Legend:** RIT, radioiodine therapy; * [36]; ** [37]; *** [24].

## Data Availability

Not applicable.
